# A Practical Treatise on the Sexual Disorders of the Male and Female

**Published:** 1900-11

**Authors:** 


A PRACTICAL TREATISE ON THE SEXUAL DISORDERS OF THE MALE AND FEMALE. New (2d) edition. By Robert W. Taylor, M. D., Clinical Professor of Venereal Diseases in the College of Physicians and Surgeons, New York. In one handsome octavo volume of 435 pages, with 91 illustrations and 13 plates in colors and monochrome. Cloth, $3.00 net. Lea Brothers & Co., Philadelphia and New York.

When Dr. Taylor put forth the first edition of this work a few years ago it was looked upon as an advance in a direction that had been neglected by the legitimate profession. The first edition was so satisfactory and became so popular that other authors entered the same field and much investigation and study followed. The author is in position to be the best authority upon this subject, and he has given us a superb work.

